# Unbiased Spontaneous Solar Fuel Production using Stable LaFeO_3_ Photoelectrode

**DOI:** 10.1038/s41598-018-21821-z

**Published:** 2018-02-22

**Authors:** Govinder S. Pawar, Asif A. Tahir

**Affiliations:** Environment and Sustainability Institute, University of Exeter, Penryn Campus, Penryn, TR10 9FE United Kingdom

## Abstract

Photoelectrochemical (PEC) water splitting to produce solar fuel (hydrogen) has long been considered as the Holy Grail to a carbon-free hydrogen economy. The PEC concept to produce solar fuel is to emulate the natural photosynthesis using man made materials. The bottle-neck in realising the concept practically has been the difficulty in identifying stable low-cost semiconductors that meet the thermodynamic and kinetic criteria for photoelectrolysis. We have fabricated a novel p-type LaFeO_3_ photoelectrode using an inexpensive and scalable spray pyrolysis method. Our nanostructured LaFeO_3_ photoelectrode results in spontaneous hydrogen evolution from water without any external bias applied. Moreover, the photoelectrode has a faradaic efficiency of 30% and showed excellent stability over 21 hours. From optical and impedance data, the constructed band diagram showed that LaFeO_3_ can straddle the water redox potential with the conduction band at −1.11 V above the reduction potential of hydrogen. We have fabricated a low cost LaFeO_3_ photoelectrode that can spontaneously produce hydrogen from water using sunlight, making it a strong future candidate for renewable hydrogen generation.

## Introduction

The current global energy consumption is approximately 14–15 TW and is expected to double by 2050. At the present time about 85% of energy provided comes from the burning of fossil fuels^1,2^. The sun is by far the largest renewable source which provides the earth with 100,000 TW of energy annually. Therefore, the energy provided from one hour of sun light illumination is equivalent to mankind’s total energy consumption for one year^1^. However due to the sporadic nature of solar energy, efficient storage devices need to be developed and employed in order to make solar energy a feasible energy source. Transportable and storable solar fuels (hydrogen) from renewable resources and efficient energy storage can only provide a sustainable pathway for true transformation to renewable energy to address the global challenge related to energy and environment.

A promising way of storing solar energy is via chemical fuels, in particular hydrogen as it is considered as a future energy carrier^3–6^. The greatest challenge is to develop a suitable technology for large scale and cost effective solar fuel production to compete with fossil fuel. One way this could be achieved is by using photoelectrochemical (PEC) water splitting which directly converts water and sunlight to solar fuel (hydrogen). Since the discovery of solar water splitting by Fujishima and Honda^7^, extensive research interest has been growing in developing efficient and stable PEC water splitting devices for a carbon free method of hydrogen generation^8–13^, however the ideal material has not been discovered.

Cost effective solar fuel generation is hindered by the semiconductor material not meeting certain essential criteria to achieve highly efficient solar to hydrogen conversion. These criteria are as follows: (i) the visible part of the solar spectrum must be absorbed for higher efficiency of hydrogen production and the band edges should ideally straddle the redox potential of water splitting, (ii) the photoexcited carriers must separate and migrate to the surface without recombination, (iii) adsorbed species must be reduced and oxidized by the photogenerated electrons and holes to produce H_2_ and O_2_^3,14^. Also for cost effective, environmental and scalability issues, earth abundant non-toxic materials should be the focus of research into new semiconductor materials.

Since Fujishima and Honda, who first demonstrated solar water splitting using TiO_2_ electrode^7^, the optimal material remains to be discovered. Many common semiconductor materials have been investigated, including TiO_2_^15^, ZnO^16^, WO_3_^17^, α-Fe_2_O_3_^18,19^, BiVO_4_^20^, metal chalcogenides^21^, Ta_3_N_5_^22^ and oxynitrides TaON^23^. Most of the stable oxide semiconductors such as SnO_2_, Ta_2_O_5_, TiO_2_, and ZnO are only photoactive in UV region due to their wide bandgap, which limits their absorption of sunlight. While small bandgap metal oxides such as α-Fe_2_O_3_, and ZnFe_3_O_4_ can harvest maximum solar spectrum, their conduction band potentials are more positive than water reduction potential, thus, they require an external bias for water splitting. Metal chalcogenides such as CdS and CdSe have ideal band structures for spontaneous water splitting but are highly unstable under water photo-oxidation conditions. Similarly, other ideal candidates such as metal nitrides and oxynitrides also suffer from photocorrosion and photo-oxidation, which limits their applications in water splitting.

A number of different p-type semiconductor materials have been developed for PEC water splitting application, such as; Cu_2_O^8^, NiO^24^, CaFe_2_O_4_^25^. These photocathode materials show good photocatalytic activity for water splitting. However, a number of issues with these materials inhibit them from performing effectively as PEC devices. Cu_2_O has been shown to have one of the highest current densities (10 mA cm^−2^) of metal oxide semiconductor materials and has attracted wide research interest, however it is highly unstable as after a few minutes the material will visibly degrade under PEC performances^26^. This high level of instability has led researchers to add protective aluminium-doped zinc oxide (AZO) and TiO_2_ layers to improve the Cu_2_O stability but this requires complicated, expensive and non-scalable techniques such as atomic layer deposition or sputtering. NiO is a highly stable material under PEC conditions, however exhibit poor photon harvesting performance due to its large band gap (3.45 eV) which only enables it to access to the UV region. CaFe_2_O_4_ has been shown to be a very promising material as it has a narrow band gap of 1.9 eV and band edges that straddle the redox potential of water (−0.6 and +1.3 V). This allows CaFe_2_O_4_ access to the visible region of the light spectrum and to be able to split water into its constituents (H_2_ and O_2_). However, it requires high fabrication temperatures (1100–1200 °C) which does not make the synthesis process very cost effective.

Recently, tremendous research efforts have been made to develop unassisted PEC system for H_2_ production and Wang *et al*. has reviewed recent progress made in unassisted PEC water splitting^27^. Among cheap visible light active and stable oxides, the bench mark PEC device with tandem structure are (i) the core-shell WO_3_/BiVO_4_/CoPi with GaAsP photovoltaic has photocurrent of 6.56 mA cm^−2^ with H_2_ evolution rate of 102 μmol/h^28^ and (ii) without photovoltaic assistance *p*-LaFeO_3_ photocathode and *n*-Fe_2_O_3_ photoanode based PEC water splitting devices have demonstrated H_2_ evolution of 11.5 μlmol/h with longest working life of 120 h^9^. Among single oxide visible PEC materials, α-Fe_2_O_3_ is one of the most investigated material, only Pt-doped α-Fe_2_O_3_:Co-Pi photoelectrode is reported to generate unassisted H_2_ from water^29^. Along with PEC water splitting, LaFeO_3_ has been involved in photocatalytic dye degradation^30,31^ and hydrogen evolution^20,21^ using powder samples. Another important fact related to LaFeO_3_ is p-type photocurrent characteristics with the potential to act as photocathode in tandem PEC cell. Stable photocathode under PEC condition is highly desirable to progress in field of solar fuel production. However, progress to improve PEC response of LaFeO_3_ photocathode is very slothful due to the lack of a sophisticated fabrication technique for high quality nanostructured thin films.

To the best of our knowledge, we report for the first time the nanostructured LaFeO_3_ photoelectrode for spontaneous hydrogen evolution from water without any external applied bias. The LaFeO_3_ photoelectrode was fabricated by spray-pyrolysis, a novel, inexpensive and scalable method, to create a stable thin film p-type semiconductor material which displays an ideal band structure with band edges saddled above and below the redox potential of water. The fabricated photoelectrode exhibits excellent PEC performance and is stable under redox water condition for more than 21 hours.

## Results and Discussion

### Material characterization

**Figure 1:**
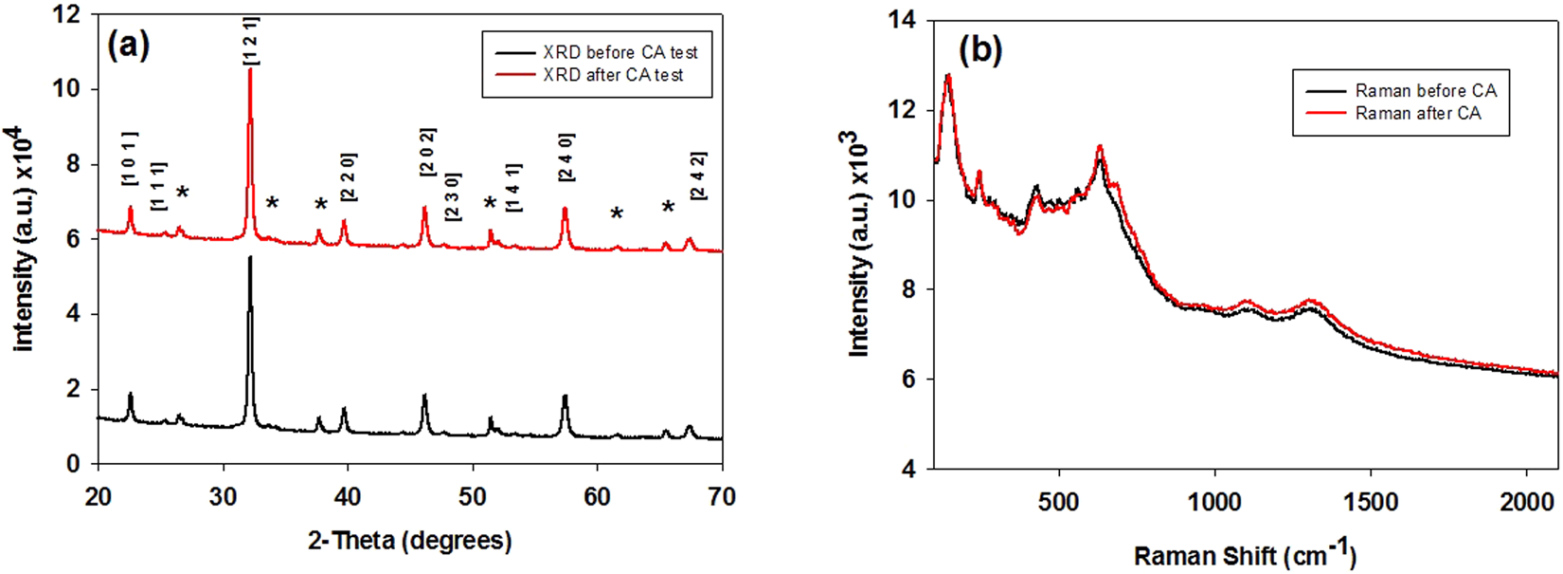
(**a**) XRD pattern of LaFeO_3_ thin film deposited on FTO glass substrate, before and after chronoamperometric test, where peaks marked with an asterisk represent FTO. (**b**) Raman shift pattern of LaFeO_3_, before and after chronoamperometric test.

LaFeO_3_ photoelectrode was prepared by spraying precursor solution at 150 °C and then annealed at different temperature from 475 °C to 625 °C with an increment of 25 °C to get single phase crystalline LaFeO_3_ material. The XRD peak patterns are shown in Figure S4. The photoelectrodes were characterised before and after chronoamperometry (CA), PEC and hydrogen evolution test to evaluate any compositional and texture change. The XRD patterns in Fig. 1a represent crystallinity and phases of LaFeO_3_ before and after tests. The XRD Peak pattern shows that the films are crystalline with LaFeO_3_ particles preferentially oriented in (121) direction. All peaks correspond to LaFeO_3_ are indexed to orthorhombic system (JCPDS 00-037-1493). No new phase is detected and there is no crystal structure change after CA, PEC and hydrogen evolution test indicating high material stability of fabricated photoelectrodes. This provides an insight into the stability of the material suggesting that it has high stability on the timescale of the experiments. The peaks marked with an asterisk distinguish peaks arising from tin oxide of FTO substrate from LaFeO_3_.

Figure 1b shows the Raman shift pattern of LaFeO_3_, which is in good agreement with previous Raman shift patterns described elsewhere^32,33^. The pattern presents the structural phase of the thin film before and after chronoamperometric test. It shows that after the chronoamperometric test there is no structural phase change in the film when compared to its fresh counterpart as the pattern overlaps that of the fresh film showing no obvious change. This data is in good agreement with the XRD pattern, providing further support that the film is highly stable and does not degrade into iron oxide and lanthanum oxide. Modes caused by the La vibrations are present below 200 cm^−1^, labelled 149 cm^−1^and assigned B_2g_. Mode 249 cm^−1^ is the lanthanum-oxygen octahedral tilt mode where it is assigned A_g_. Modes between 400–450 cm^−1^ are correlated to oxygen octahedral bending vibrations, assigned B_3g_, and modes above 500 cm^−1^ are oxygen stretching vibrations^33^. The peaks at 1128 and 1317 cm^−1^ can be attributed to the two photon scattering, however in the literature it is hotly debated whether the peak at 637 cm^−1^ is due to impurity or two photon scattering^32^. However considering the XRD pattern, it is most likely due to two photon scattering as XRD shows a single phase of LaFeO_3_. No other peaks corresponding to lanthanum oxide or iron oxide was found which is in good agreement to the XRD pattern.

**Figure 2:**
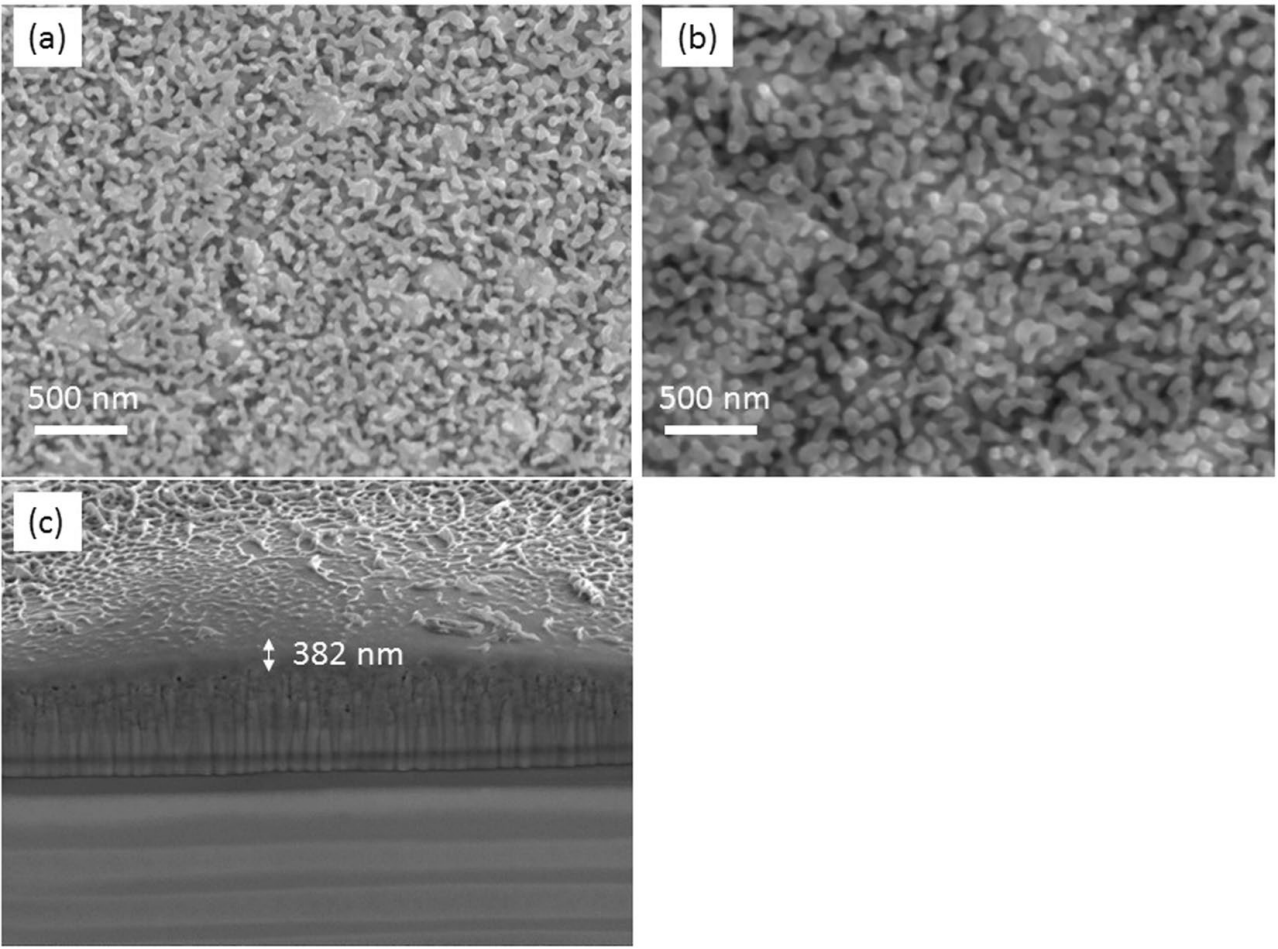
(**a** and **b**) Top view SEM of LaFeO_3_ thin film before and after chronoapeometric test and (**c**) FIB-SEM cross section of LaFeO_3_ photoelectrode.

Figure 2a shows the top view SEM image of the LaFeO_3_ photoelectrode before the chronoamperometric test. Here it is observed that the nanostructure of the material has excellent compact uniformity and well connected crystal grains in a coral like structure, post-annealing at 550 °C. Figure 2b shows the top view SEM image of the film after the chronoamperometric test. We can clearly note that the film retains its uniformity and good interconnection between the crystal grains. Figure 2c shows the FIB-SEM cross section of the film with thickness of 382 nm. In addition, Figure S1 shows the EDS of LaFeO_3_ indicating the lanthanum, iron and oxygen peaks. This further confirms the composition of the materials, which aligns with the XRD and Raman patterns. Figure S2 shows that the lanthanum and iron are homogenously mixed in the LaFeO_3_ film, by element mapping from top view EDS characterization, further emphasizing the uniformity of the film.

### Photoelectrochemical (PEC) analysis

**Figure 3:**
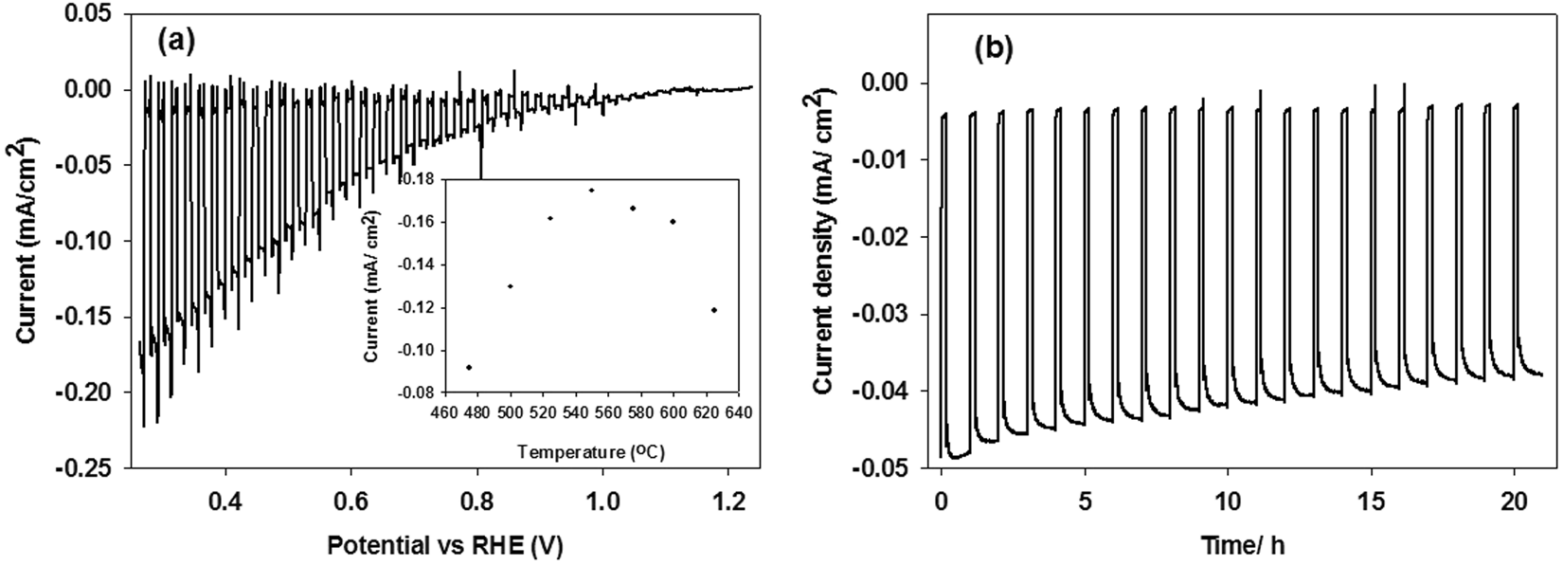
(**a**) J-V characteristics of LaFeO_3_ under chopped illumination in a 0.1 M pH 13 electrolyte. (**b**) Chronoamperometry test of LaFeO_3_ under chopped illumination in a 0.1 M pH 13 electrolyte at −0.3 V vs Ag/AgCl.

The PEC performance of LaFeO_3_ was performed in 0.1 M aqueous NaOH (pH 13) solution by illuminating the photoelectrode from the electrolyte side, where the light was chopped every 0.01 V. The photocurrent density (*J*) is plotted against bias potential (*V)* as shown in Fig. 3a. The steady state photocurrent onset potential estimated from the *J-V* plot was at 1.2 V vs. RHE. The photocurrent density rises up to approximately 0.16 mA cm^−2^ at 0.26 V vs. RHE, while no clear dark current is observed between the measured potential ranges. Due to the compact nature of the film it will be difficult for the electrolyte to penetrate deep into the film, thus we have a relatively low photocurrent. The large spikes during each chop is due to transient electrons generated undergoing recombination with their respective hole, as the accumulated charges do not have enough time to interact with the electrolyte before/after each rapid chop. This recombination effect can be seen when comparing Fig. 3a *J-V* plot to the *J-V* plot of Figure S3, where the light was chopped at a slower rate every 0.5 V. As the light is chopped at a slower rate it allows the accumulated charges enough time to interact with the electrolyte solution allowing the charges to separate, hence we do not observe the spikes^34^. This indicate a slow charge transfer dynamics which need to improve and investigate further to enhance the efficiency of LaFeO_3_ photoelectrodes.

To determine the best fabrication conditions for LaFeO_3_ photoelectrode, annealing temperature, spray amount and time has been optimised. The annealing temperature was found to be an important factor which directly affected the photocurrent density of LaFeO_3_ photoelectrodes. The dependence of photocurrent density on annealing temperature is shown as inset in Fig. 3a while XRD peak patterns are given in Figure S4 and the effect of annealing temperature on *J-V* curve is given in Figure S5. The photocurrent density increases with the increase of annealing temperature and reaches the optimum level at 550 °C. With further increase of the deposition temperature, the photocurrent of LaFeO_3_ photoelectrodes decreases significantly. XRD shows that at low temperature regime, the decomposition was not completed and the XRD peak for LaFeO_3_ was not detected at 475 °C. The LaFeO_3_ XRD peak starts appearing at 500 °C and becomes more crystalline with the increase in temperature. The low photocurrent at lower (<550 °C) and high (>550 °C) temperature regime can be due to incomplete decomposition and sintering of film respectively which can create defects, dislocations and kink sites in the film. These dislocations and kink sites may act as the recombination centres for the photogenerated electron-hole pairs and consequently showing a poor photocurrent density.

The photocurrent density varied with amount of spray solution/time. The amount of spray solution/time was directly correlated to the photoelectrode thickness (data not shown). As expected the photoelectrodes correspond to short spray times are relatively thin whereas the electrodes associated with long spray times are thick. The photoelectrode deposited using 3 ml spray solution shows lowest photoresponse, which increases with increase in spray time and reached to maximum at 5 ml spray amount and then decrease. The *J-V* characteristics are shown in Figure S6. The photoelectrode which showed the maximum photocurrent density had a thickness of 382 nm and corresponds to 5 ml spray solution/5 minutes of spray time. For electrodes with higher thickness the photogenerated electrons require to travel more before collecting at the FTO substrate (here the electrodes were illuminated from the electrolyte side) depending on the thickness of photoelectrode. The electron transport within LaFeO_3_ is significantly slow as evident from high recombination spikes in faster chopped *J-V* (Fig. 3a). The slow and trap dominant charge transport mechanisms within nanostructured matrix have been already reported in other metal oxide semiconductor systems^35^.

A chronoamperometric test of the LaFeO_3_ photoelectrode was conducted to determine its stability. Figure 3b shows the results from the test where it was carried out in an aqueous 0.1 M (pH 13) electrolyte solution at a constant potential of −0.3 V over a period of 21 hours under chopped illumination, where the film was illuminated for 45 minutes and in dark for 15 minutes every hour for 21 hours. A small gas bubble accumulation on the surface of the electrode was also observed during the test. From the graph it is observed that over the 21 hour period the photoelectrode remains photoactive giving a very stable response. However, a very slight gradual decrease in current density is observed which could be due to gas bubble accumulation. As the gas bubbles form it causes a shading effect on the photoelectrode as they tend to stick on the electrode surface, decreasing the effective area, increasing the interfacial electric resistance^36^, hence we see the slight gradual decrease in current density. The slight decrease in photocurrent start after an hour which is in agreement with H_2_ evolution. Another reason for the decrease of the photocurrent could be due to the space charge accumulation on the surface of the film^37^. As the reaction is taking place the build-up of surface charge will create a barrier which will prevent the charges being taken up by the electrolyte, as seen in the slight drop in photocurrent. These effects can have an adverse effect on the hydrogen evolution efficiency as less electrolyte is able to penetrate the film to be available for effective charge separation. This can therefore cause a reduction in hydrogen evolution efficiency over time.

### Optical and Electrochemical Measurements

**Figure 4:**
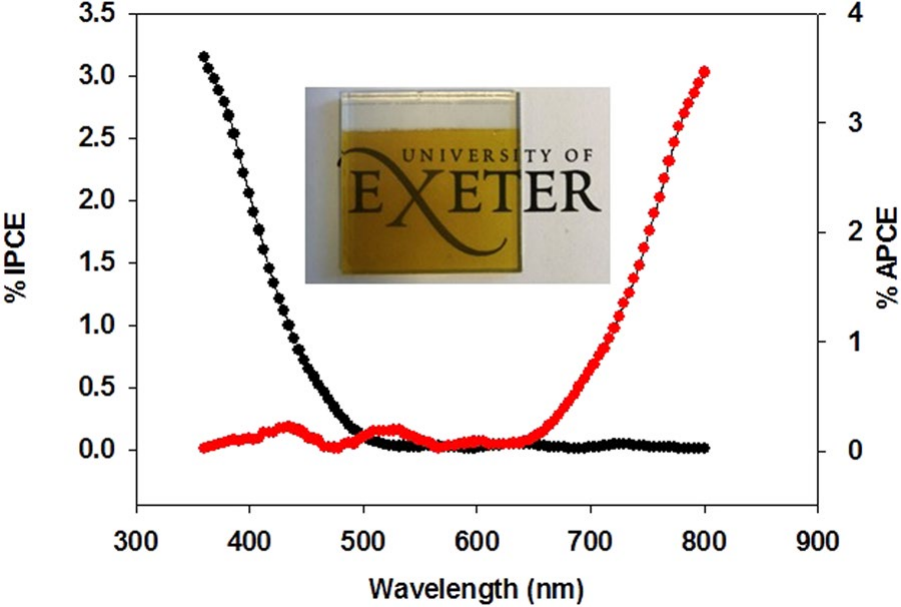
IPCE and APCE spectra for the nanostructured LaFeO_3_ thin film.

Another factor causing the low photocurrent may be due to the incident photons hitting the film not being sufficiently absorbed. This may be due to the film being very thin. An incident photon to electron conversion efficiency (IPCE) measurement was recorded at 0.26 V vs RHE for the LaFeO_3_ photoelectrode using an aqueous solution of 0.1 M NaOH. This measurement helps to determine how efficiently the photons were being converted to current, shown in Fig. 4. The IPCE threshold exists at about 520 nm, where the maximum efficiency of 3.2% is obtained at 350 nm. The relatively low IPCE exhibited by the LaFeO_3_ electrode may be due to the combined factors of weak light absorption, hole-electron recombination rate^38^ and very thin film. The IPCE increased substantially from 400 nm indicating that absorbed photons of different energies have been successfully converted to photocurrents. However, the IPCE value is higher for short wavelength and decrease rapidly over 400–600 nm region compares to absorption spectrum. The IPCE near bandgap at long wavelengths is lower, which is a common characteristic of iron based photoelectrode^29^. An analysis of IPCE and absorption data allows us to calculate the absorbed photon to electron conversion efficiency (APCE). The APCE is determined from the IPCE and light harvesting efficiency (LHE) using the following equations^39^:

1 $$LHE=1-\,{10}^{-A}$$

2 $$APCE=\frac{IPCE}{LHE}$$

where *A* is the absorbance at certain wavelengths and IPCE is determined experimentally. The APCE spectra overlaps with the IPCE spectra over the entire spectral range. The APCE shows a maximum efficiency of 3.5% which is in good correlation to the IPCE, suggesting that the quality of the film is good however it has very weak light absorption capabilities which may account for the low photocurrent (seen in Fig. 3a) and low faradaic efficiency. In principle, it is possible to increase the thickness of active layer to absorb more photons, thus achieving a higher photocurrent. However, increasing the thickness will increase the internal resistance of the device^40^.

**Figure 5:**
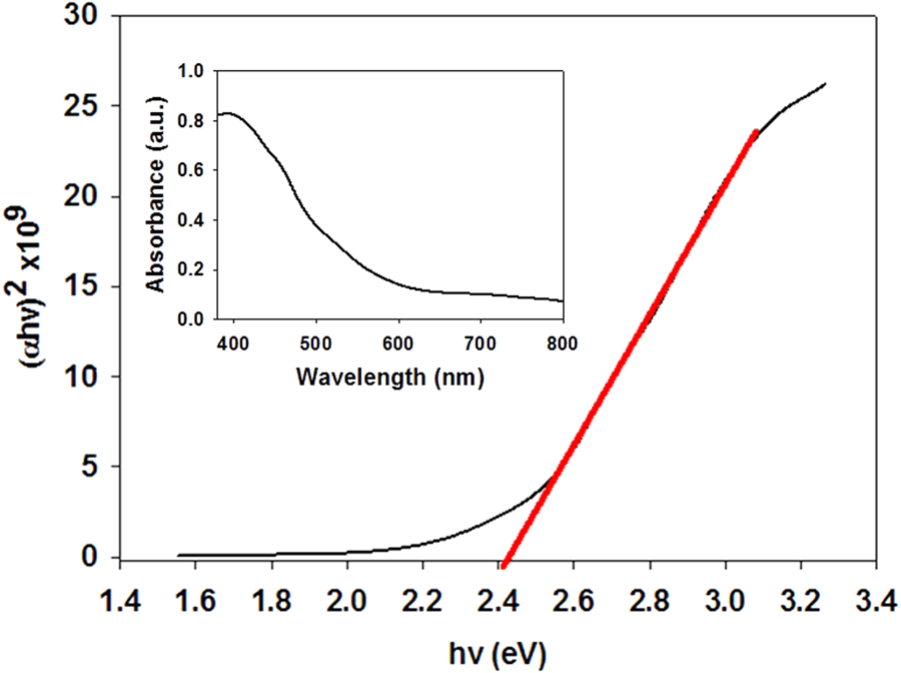
Tauc plot of LaFeO_3_ showing the band gap energy (E_g_) while inset shows Absorbance spectra of LaFeO_3_.

Optical absorbance measurements were taken for LaFeO_3_ to help determine the materials band gap. Figure 5 presents the material’s absorbance measurement for the LaFeO_3_ photoelectrode between the ranges of 350–800 nm. The low absorption and IPCE is attributed to very thin and semi-transparent nature of film (shown as an interest in Fig. 4). By using this data the band gap energy (E_g_) of the LaFeO_3_ film can be determined using Tauc plot^41^, by plotting (α*hv*)^1/n^ vs. *hv* and the band gap is determined by the x-axis intersect, where α is the absorption coefficient and *hv* is determined by 1240/wavelength. *n* is 1/2 for direct allowed transitions or 2 for indirect allowed transition. In our case *n* value of 1/2 was selected. α is defined by Beer-Lambert’s law and can be determined by the following equation^42^:

3 $$\alpha =\frac{2.303\,x\,A}{{\rm{d}}}$$

where *A* is absorbance at wavelength (λ) and d is film thickness. Figure 5 shows that LaFeO_3_ photoelectrode has a direct band gap of 2.4 eV, shown where the red line intersects the x-axis. This band gap value for LaFeO_3_ falls in line with other works conducted^43^. The narrow band gap illustrates that the material is capable of absorbing visible light.

**Figure 6:**
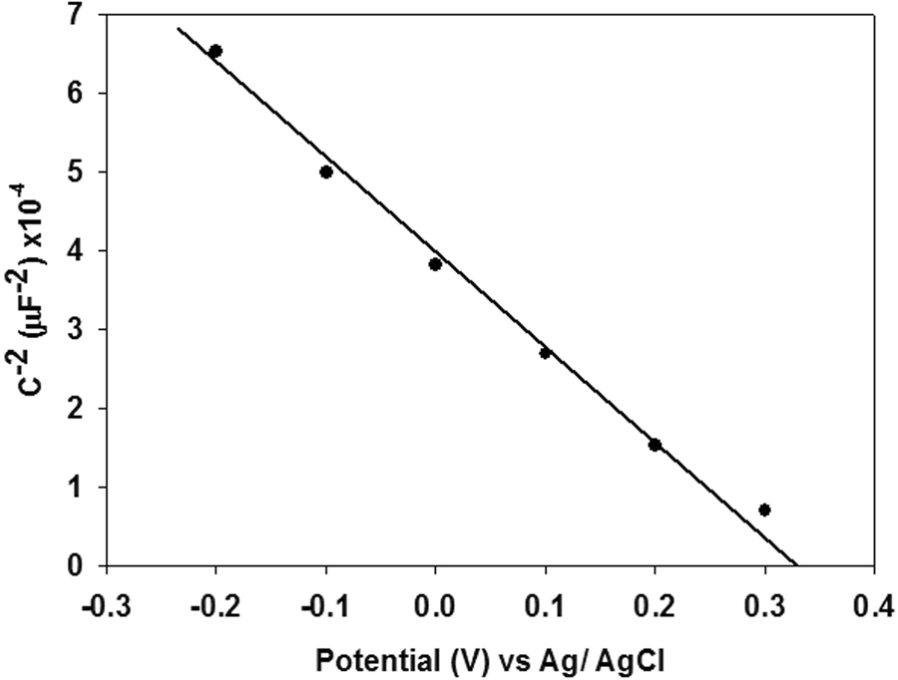
Mott-Schottky plot of LaFeO_3_ thin film obtained from impedance measurement in the dark in pH 13.

**Figure 7:**
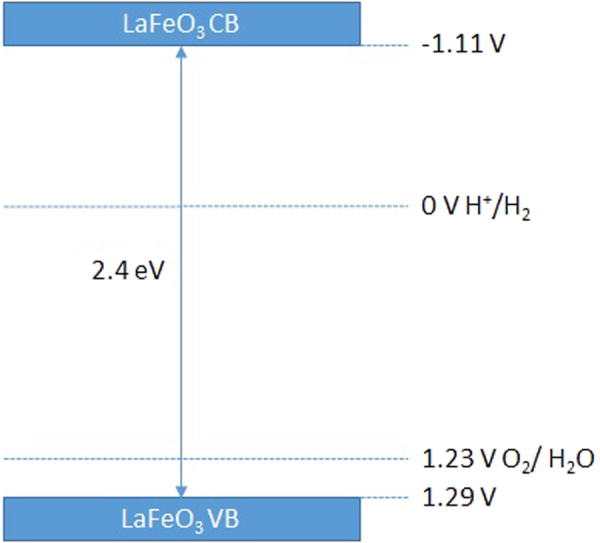
Band diagram of LaFeO_3_ vs RHE.

Electrochemical impedance spectroscopy measurement (EIS) was conducted in order to determine the semiconductors flatband potential. Impedance data were fitted in ZView to a Randles circuit to extract the capacitance. The Mott-Schottky plot showed p-type characteristics of our LaFeO_3_ film. Flat band potential was extracted using the Mott-Schottky equation for p-type semiconductors:

4 $${(\frac{1}{C})}^{2}=\,\frac{2}{\varepsilon {\varepsilon }_{0}{A}^{2}e{N}_{D}}(V-\,{V}_{{\rm{fb}}}-\,\frac{{k}_{{\rm{B}}}T}{e})$$

where *C* is capacitance, *e* is the electronic charge, *ε*_*r*_ is the relative permittivity of materials, *ε*_0_ is the permittivity of vacuum, *N*_*A*_ is the carrier concentration, *k* is the Boltzman constant, *T* is the absolute temperature, *A* is the area of electrode, *V* is the applied potential and *V*_*fb*_ is the flat band potential^44^. *V*_*fb*_ of LaFeO_3_ was determined through a linear fit in the linear region of the Mott-Schottky plot and were calculated to be 0.328 V vs Ag/AgCl (Fig. 6). The measured flat band can be converted to the RHE scale using the Nernst equation^45^:

5 $${E}_{{\rm{RHE}}}={E}_{\mathrm{Ag}/\mathrm{AgCl}}+0.059{\rm{pH}}+E{^\circ }_{Ag/AgCl}$$

where *E*_RHE_ is the converted potential vs. RHE, *E°*_*Ag/AgCl*_ = 0.197 V at 25 °C and *E*_Ag/AgCl_ is the experimentally measured potential against Ag/AgCl. The flat band was determined to be 1.29 V vs. RHE. The flat band potential is in good agreement with the photocurrent onset potential measured from J-V curve. Using a varied Mott-Schottky equation^46^ (equation 6) the carrier density can be calculated.

6 $${{C}_{SC}}^{-2}=(2/e{\varepsilon }_{0}\varepsilon {N}_{D})({V}_{m}-{V}_{FB}-kT)$$

where *e* is the electronic charge, *ε*_0_ is the permittivity of vacuum, *ε* is the dielectric constant, *N*_D_ is the carrier density, *V*_m_ is the measured potential and *V*_fb_ is the flat band potential. 6 × 10^3^ value is used as the dielectric constant^47^. The carrier (hole) density was calculated to be 15.27 × 10^18^ cm^−3^. With the flatband and band gap values determined, we can construct a relative band alignment of the LaFeO_3_ material to the waters redox potentials, as shown in Fig. 7. The valance band and the conduction band is shown to be straddling the redox potential of water with a narrow band gap. This suggests that the material is able to generate hydrogen using the visible part of the spectrum, as the conduction band is well above the reduction potential of hydrogen. This data shows that the LaFeO_3_ photoelectrode is able to produce hydrogen from water.

### Hydrogen Evolution Measurement

**Figure 8:**
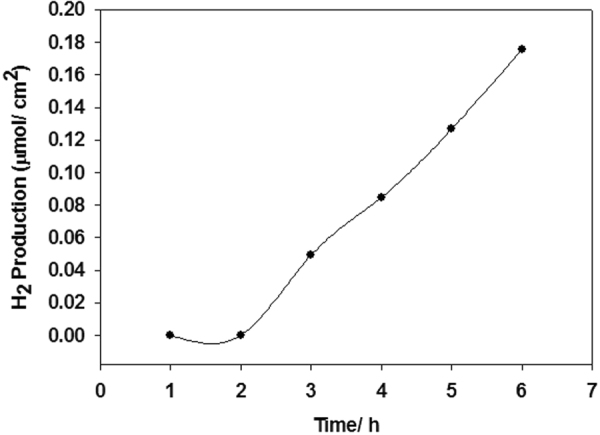
Hydrogen evolution test of LaFeO_3_ in an aqueous 0.1 M NaOH.

Figure 8 shows the hydrogen production performance of the LaFeO_3_ photoelectrode in an aqueous 0.1 M NaOH solution under a constant illumination. The water splitting test was conducted in a custom made glass reactor vessel (Figure S7) with an attached fused silica viewport. The LaFeO_3_ working electrode and Pt counter electrode were connected by a single looped wire, without any external bias being applied. Hydrogen was being produced spontaneously during the water splitting test during the first 6 hour cycle where the photoelectrode generated 0.18 μmol/cm^2^ of hydrogen after 6 hours, with a faradaic efficiency of 30%. It then underwent a second cycle of water splitting test to determine if the electrode was re-usable and how much the performance varied. After a further 6 hours illumination, the LaFeO_3_ thin film generated 0.08 μmol/cm^2^ of hydrogen (Figure S8). This provides additional evidence that the film is re-useable, although the amount of hydrogen produced is almost halved. In addition, it should be noted that the low amount of hydrogen produced and low faradaic efficiency can be attributed to the low photocurrent generated. The low photocurrent correlates with low hydrogen produced as an insufficient amount of electrons are being generated to produce a high amount of hydrogen, as seen in the *J-V* curve. Also, the electrons which are being generated are not all being converted to hydrogen as we see a low faradaic efficiency. This can be due to the electrolyte not effectively penetrating the film due to its compact morphology and/or gas bubbles forming on the photoelectrode where they tend to stick on the electrode surface, decreasing the effective area, increasing the interfacial electric resistance and thus increasing the losses^36^. After the second cycle of water splitting test, the amount of hydrogen produced further decreases. Again this can be because of the gas bubbles accumulating onto the film surface preventing causing a shading effect, inhibition of effective charge separation and/or space charge layer accumulated onto the film surface. This in turn hinders charge separation, lowering efficiency.

## Conclusion

In summary, we have developed a stable p-type LaFeO_3_ photoelectrode with a coral like nanostructure by a novel and inexpensive spray pyrolysis technique with a post annealing step, which yields a photocurrent density of 0.16 mA/cm^2^ at 0.26 V vs. RHE. Chronoamperometric studies showed that the LaFeO_3_ film provides a stable p-type response over a 21 hour period. Optical and impedance data showed that the material is able to straddle the redox potential of water, with the valance band at 1.29 V and conduction band at −1.11 V, and a bandgap of 2.4 eV. IPCE studies revealed that the photoelectrode had an APCE of 3.5%. Water splitting test was conducted in a custom made reactor vessel, where the working electrode and Pt counter electrode was connected by a single looped wire, without any external bias being applied. This in turn yielded 0.18 μmol/cm^2^ hydrogen after six hours during the first cycle with faradaic efficiency of 30%. To the best of our knowledge this is the first time hydrogen has been produced spontaneously during a water splitting test without any external bias being applied using LaFeO_3_ photoelectrode as a single material. These findings demonstrate that LaFeO_3_ is a potential candidate to act as a photoelctrode for unassisted PEC water splitting to generate solar fuel (hydrogen) cost effectively. However further work is required to investigate and improve slow charge carrier dynamics and low light absorption challenges of LaFeO_3_ photoelectrodes.

## Method

### Fabrication of LaFeO_3_ (LFO) Substrate by Spray Pyrolysis

Iron (III) nitrate nonahydrate (1 mmol) was dissolved in methanol and 20 ml 30% solution of NH_3_ in water was added to generate precipitate of iron hydroxide. The precipitate was collected by centrifuge and washed two times with de-ionized water. Iron hydroxide was then dissolved 0.1 ml of Trifluoroacetic acid (99%) in 25 ml methanol and Lanthanum (III) iso-propoxide (1 mmol) was added. Once fully dissolved, the solution was used for spray pyrolysis.

Fluorine doped tin oxide (FTO) glass substrates were cleaned ultrasonically (prior to spray pyrolysis) by ethanol, iso-propanol and acetone 15 minutes each, in that order, and then washed with de-ionized water to remove any remaining organic substances and dried with compressed air. Once cleaned the FTO glass substrate was placed in the centre of a hot plate at 150 °C. The spray system comprised of a syringe pump system (New Era Pump System NE-1000), an ultrasonic atomizer nozzle (Sonozap) 1 mm diameter and a vortex attachment. 5 ml of the precursor solution was sprayed on to the FTO at a rate of 1 ml min^−1^ assisted with compressed air at a rate of 6 L min^−1^, which is passed through the vortex attachment to generate a large plume of aerosol to get a uniform coverage on the FTO. After the completion of spray, the films were further annealed at 550 °C for 3 hours in air.

All data generated or analysed during this study are included in this published article (and its Supplementary Information files).

## Electronic supplementary material

Supplementary Information
