# Supplementary material for: Unbiased Spontaneous Solar Fuel Production using Stable LaFeO3 Photoelectrode
Source: Sci Rep. 2018 Feb 22;8:3501. doi: 10.1038/s41598-018-21821-z (PMC5823922; doi:10.1038/s41598-018-21821-z)
Supplement: Supplementary file 1 — Supplementary Information [file 41598_2018_21821_MOESM1_ESM.docx]

**Unbiased Spontaneous Solar Fuel Production using Stable LaFeO_3_ Photoelectrode**

Govinder S. Pawar and Asif A. Tahir*

**Instrument details**

**Material Characterization**

The material phase composition was determined using a Bruker D8 Advance X-ray diffractometer (Cu Kα irradiation, 40kV/ 40mA, 0.02 ^o^2θ step and a scan time of 3 seconds per step) in the range of 20-70 ^o^2θ. The morphology and composition of the thin film was characterized using a high resolution scanning electron microscope (SEM, HITACHI S3200N) coupled with an energy dispersive spectroscopy (EDS, Oxford instrument elemental analysis). Film thickness was characterized using focus ion beam scanning electron microscope (FIB-SEM, NOVA NanoLab FEI 600 dual beam FIB SEM). Diffuse reflectance spectra was acquired using a spectrophotometer (PerkinElmer lambda 1050 with 150mm integrated InGaAs sphere). The incident photon to electron conversion efficiency (IPCE) was obtained by measuring the incident photon flux using a 75 W xenon lamp connected to a monochromator (TMc300, Bentham Instruments Ltd., Berkshire, UK). The light was calibrated using a silicon diode. Photocurrent spectra were measured at -0.7 V using a combination of a lock-in amplifier (Bentham Instrument Ltd., Berkshire, UK) and a custom built potentiostat. The Raman shift pattern was obtained using the Renishaw RM1000 (Renishaw plc, Gloucestershire, UK) with a 532 nm Ar ion-laser.

**Electrochemical Characterization**

All electrochemical experiments were carried out in a standard 3 electrode system, composed of a working electrode, Pt wire as counter electrode and a reference electrode of Ag/AgCl in saturated KCl. All data were collected by a potentiostat (SP 200, BioLogic Science Instruments). The working electrode potential versus the Ag/AgCl reference electrode used in all experiments was converted into the reversible hydrogen (RHE) electrodeusing equation: *V*_RHE_ =*V*_Ag/AgCl_ + 0.197 V + 0.059 V *pH.

The photoelectrochemical (PEC) performance of LaFeO_3_ photoelectrode was measured in 0.1 M NaOH aqueous solution (pH 13) under chopped light illumination using a 100 W ozone free Xenon lamp equipped with an AM 1.5 filter (Oriel LCS-100, Newport). The light intensity was 1 sun illumination (100 mW cm^-2^). A liner sweep voltammetry (LSV) was scanned in the positive to negative direction between the ranges of +0.3 V to -0.7 V.

Mott-Schottky analysis was carried out using electrochemical impedance spectroscopy (EIS) in the dark in 0.1 M NaOH (pH 13). The impedance spectra were acquired at frequencies from 3 MHz to 10 mHz in the potential range of -0.3 V to +0.6 V.

Chronoamperometric (CA) measurements of LaFeO_3_ were conducted over a period of 21 hours under 1 sun illumination with periodic chopping. The sample was subjected to illumination conditions of 45 minutes and dark conditions for 15 minutes. This was carried out in 0.1 M NaOH (pH 13) in a standard 3 electrode system in ambient atmosphere and temperature. A constant current of -0.3 V was maintained over the measurement period.

Gas chromatography (GC) measurements were carried out using a manual injection GC system (PerkinElmer Clarus 580) using a molecular sieve (PerkinElmer) and a PDD detector with an argon flow of 28 ml min^-1^. A custom made glass reactor vessel (Figure S7) with an attached fused silica viewport containing 0.1 M NaOH (pH 13) with a dead space of 100ml was purged with argon for 2 hours with gentle heating and stirring to remove atmospheric air from the system. The sealed vessel contained the working LaFeO_3_ electrode connected to a Pt mesh by a single outer wire and was subjected to light illumination for the water splitting reaction. GC measurements were taken every 1 hour.

**
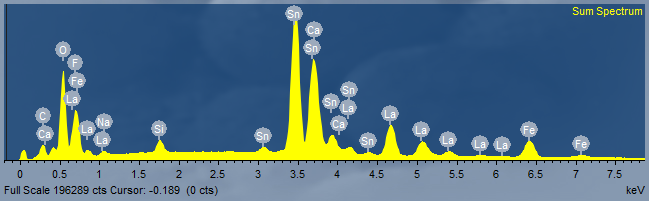
**

**Figure S1** EDS of LaFeO_3_ showing peaks of lanthanum, iron and oxygen.

**Fe La**

**
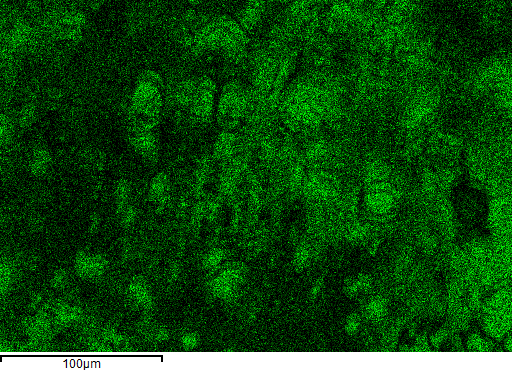

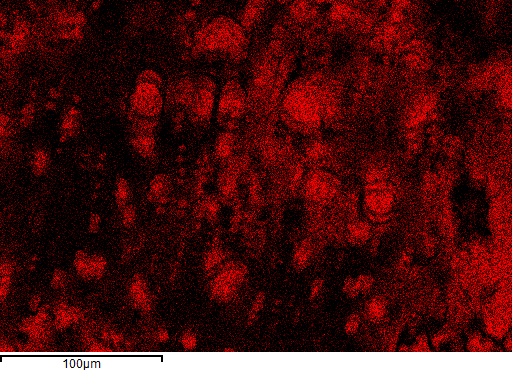
**

**Figure S2** EDS of LaFeO_3_ showing the distribution of iron and lanthanum in the film


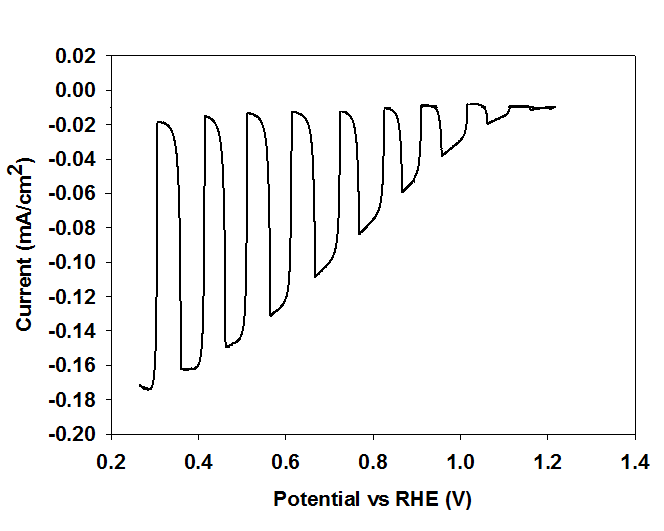


**Figure S3** J-V characteristics of LaFeO_3_ under chopped illumination, where each chop was done every 0.5V, in a 0.1M NaOH pH 13 electrolyte.


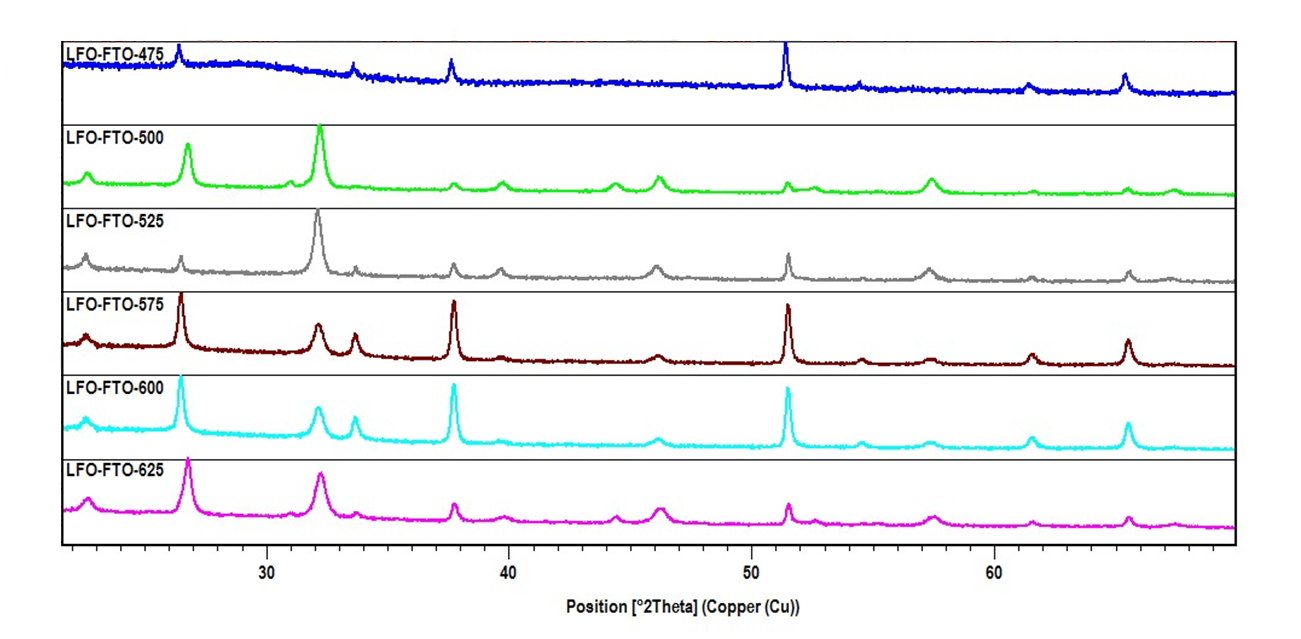


**Figure S4** XRD pattern of LaFeO_3_ at various annealing temperatures

**Figure S5** J-V characteristics of LaFeO_3_ at various annealing temperatures under chopped illumination in a 0.1M NaOH pH 13 electrolyte.

**Figure S6** *J-V* characteristics of different film thickness under chopped illumination in a 0.1M NaOH pH 13 electrolyte.


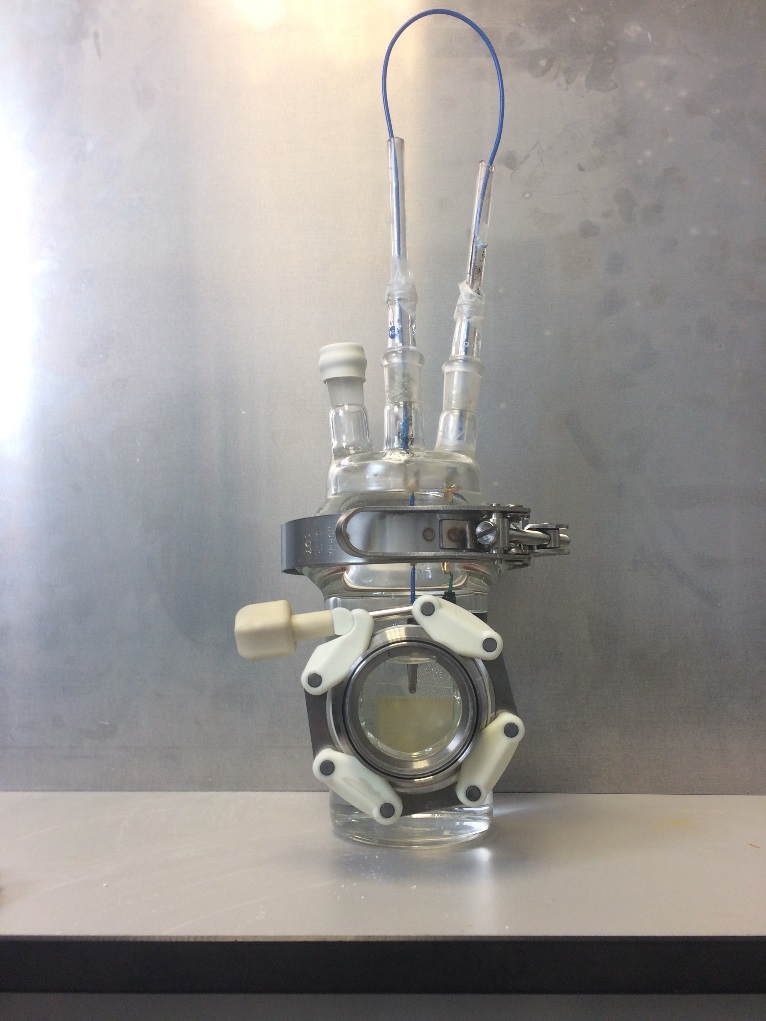


**Figure S7** Custom made reactor vessel for hydrogen evolution test with a fused silica quartz viewport. Working electrode and counter electrode connected by a single wire.

**Figure S8** Solar hydrogen production cycle 2 in 0.1M NaOH after further 6 hours illumination
